# Transductive Learning From a Transformer Foundation Model Improves the Taxonomic Classification of eDNA Metabarcodes

**DOI:** 10.1111/1755-0998.70204

**Published:** 2026-11-11

**Authors:** Simon Bettinger, Auguste Verdier, Morgane Bruno, Stéphanie Manel, Alexis Joly, Maximilien Servajean, Alice Valentini, Rodolphe Devillers, David Mouillot

**Affiliations:** ^1^ MARBEC, Univ Montpellier, CNRS, IFREMER, IRD Montpellier France; ^2^ CEFE, Univ Montpellier, CNRS, EPHE‐ PSL University, IRD Montpellier France; ^3^ Institut Universitaire de France Paris France; ^4^ Inria, Univ Montpellier, LIRMM, CNRS Montpellier France; ^5^ SPYGEN Le Bourget‐du‐Lac France; ^6^ Espace‐Dev, IRD, Univ Montpellier, Univ Réunion Montpellier France

**Keywords:** deep learning, DNABERT, environmental DNA, fish biodiversity, metabarcoding, transformer models

## Abstract

Environmental DNA (eDNA) has emerged as a powerful and cost‐effective biodiversity monitoring tool, providing comprehensive and non‐invasive taxonomic inventories. However, eDNA‐based monitoring is strongly dependent on the completeness of genetic reference databases, which varies greatly among regions and taxa. Since completing gaps at the species level is unrealistic in the short term, an alternative is to infer coarser taxonomic assignments for sequences belonging to species not yet in reference databases. To address this challenge, we introduce *BouillaBERT*, a novel deep learning method tailored for the operational classification of eDNA sequences from unknown genera. *BouillaBERT* leverages data augmentation, transfer and transductive learning to overcome the small size and imbalance of eDNA reference databases for fishes. We implement and train the model across four commonly used fish eDNA markers and compare its performance with lowest common ancestor (LCA) alignment methods and existing deep learning approaches. *BouillaBERT* outperforms all tested LCA alignment and deep learning methods in recall for the assignment of unknown sequences across a range of commonly used fish eDNA primers, while achieving comparable or higher precision. *BouillaBERT* can be rapidly re‐trained to fit a custom sample set or used as‐is, enabling more consistent biodiversity assessments across undersampled regions or taxa.

## 1 Introduction

Biodiversity is undergoing unprecedented decline, with around 28% of species being threatened worldwide (IUCN 2025), and one million facing extinction due to anthropogenic pressure (IPBES 2019). To face these threats, effective conservation policies require reliable, non‐extractive, long‐term biodiversity monitoring programmes that provide information on species geographic distributions and the composition of local communities (Gonzalez et al. 2023). Beyond biodiversity loss, communities have also experienced marked species turnover for decades, driven by the balance between local species extirpations and colonization events (Dornelas et al. 2019). On the one hand, such shifts in species composition alter ecosystem processes and functioning by modifying energy fluxes, trophic relationships and productivity (Dee et al. 2023; O'Gorman et al. 2023). On the other hand, these shifts can imperil ecosystem services and impose high societal costs on human populations that depend on biodiversity stability (Chapin III et al. 2000; Storch et al. 2022). Consequently, ecological monitoring should also focus on the composition of communities, beyond the mere species richness or individual populations (Dornelas et al. 2019).

In many ecosystems, such community‐level information is still challenging to obtain. Sampling approaches differ widely among species and habitats (Bowler et al. 2025), while integrating measures from multiple sources often proves challenging (Isaac et al. 2020). In the marine realm, standardized data are especially logistically and methodologically difficult to collect given the vast, harsh and inaccessible environments but also the mobility or elusivity of some species (Klaassen et al. 2025). Traditional fishing surveys remain destructive and ethically problematic (Trenkel et al. 2019) notably in at‐risk environments like coral reefs (Murphy and Jenkins 2010) or protected areas (McGeady et al. 2023). Additionally, the species inventories they provide are biased by mesh size and net opening (Le Joncour et al. 2024). Video monitoring is less intrusive and highly replicable across the ocean (Letessier et al. 2024), but depends on good visibility and mainly targets conspicuous species and scavengers in the case of baited sampling (Holmes et al. 2013; Murphy and Jenkins 2010). Underwater visual surveys by human operators are non‐invasive and cost‐effective (Edgar et al. 2020), but are limited to surface waters, cannot operate in turbid environments or in the high sea, and are biased for top predators like sharks that avoid divers (Boussarie et al. 2018).

Over the past decade, environmental DNA (eDNA) metabarcoding has emerged as a powerful alternative for biodiversity monitoring, especially in aquatic environments (Altermatt et al. 2025; Çevik and Çevik 2025; Takahashi et al. 2023). Although the method is cost‐effective and non‐invasive, eDNA has been shown to provide comprehensive and robust biodiversity inventories (Iacaruso et al. 2025), including for rare and elusive species (Boussarie et al. 2018; Perry et al. 2024). Yet, the accurate taxonomic assignment of eDNA metabarcodes is critical in ecosystem monitoring and management but still challenging to achieve since the completeness of the genetic reference databases (De Santiago et al. 2025; Schenekar et al. 2020) varies widely among regions and taxa (Marques et al. 2021). For instance, while the Northwestern Mediterranean fish database for the Teleo marker is 75% complete (Dalongeville et al. 2022), supporting the widespread use of eDNA metabarcoding in monitoring campaigns even for at‐risk species like elasmobranchs (Pichot et al. 2025), tropical waters remain poorly documented. For instance, reference sequences are missing for up to 66% of fish species in the Western Coral Triangle (Juhel et al. 2020). This is particularly problematic as tropical regions are hotspots of vulnerable species, with more than 57% of threatened species (IUCN VU/EN/CR) remaining unreferenced in the Western Coral Triangle (Marques et al. 2021). Incompleteness is also high in ecosystems where species sampling is challenging, such as the deep sea, and for taxonomic groups that remain poorly represented, like many cryptobenthic fishes (Duhamet et al. 2023). While accelerating climate change and biodiversity loss urgently require reliable biodiversity monitoring, filling those gaps in genetic reference databases is time‐consuming and seems unrealistic in the short term.

Incidentally, reference sequences are more likely to be missing for lower taxonomic ranks. In the Western Coral Triangle, while only 60% of fish genera are represented by at least one genetic sequence, up to 89% of families are covered in a genetic reference database (Marques et al. 2021). This gap can be exploited by assigning sequences of missing species or genera to a coarser rank, for which genetic reference is available. Consequently, an alternative approach to the completeness of genetic reference databases is to infer taxonomic assignments for sequences belonging to taxa where finer taxonomic reference is unavailable. Traditional alignment‐based methods can address this challenge since they rely on close matches with existing known sequences from reference databases (Mirdita et al. 2021) but cannot combine patterns across multiple, partially related sequences. Recently, the development of deep learning methods enables the detection of intricate patterns across training sequences and the generalization towards unknown sequences. Among the first applications of such approaches using artificial intelligence, BERTax (Mock et al. 2021) adapted a transformer‐based language model (BERT) to the taxonomic classification of DNA sequences, which are considered as text. BERTax has demonstrated that deep learning can indeed outperform traditional bioinformatics approaches, especially on the task of classifying sequences from unknown genera. However, BERTax was designed for the classification of long sequences (i.e., 1500 nucleotides), and its performance requires training on a very large and specific dataset. In addition, while BERTax provides genus predictions for its ‘similar dataset’ task, its assignments of unknown sequences are restricted to high taxonomic levels (i.e., phylum and superkingdom) that are too broad to guide biodiversity conservation and management (Mueller et al. 2013) where identification is required at the family or finer taxonomic levels (Condachou et al. 2023). In addition, relevant eDNA primers used in ecology and environment produce metabarcodes that are only a few tens to a few hundreds of nucleotides long (Zhang et al. 2020), and very specific (Collins et al. 2019; Jo et al. 2017). Genetic reference databases for these markers are comparatively small, as a majority of species are only represented by a single instance of the target marker, if present at all. In addition, unequal sampling of genetic material across regions and taxa (Manel et al. 2020) makes such databases very imbalanced for these eDNA markers (Marques et al. 2021). This in turn makes amplicon taxonomic assignment by deep learning models very challenging.

Here, we aim to overcome these limitations by proposing a novel taxonomic classification framework for the assignment of unknown eDNA sequences through the coupling of a transductive learning approach and a transformer foundation model (DNABERT‐2) that we adapt to the constraints of eDNA metabarcodes. The transductive learning approach was first proposed by Vapnik (1998), where the model exploits the structure of both training and testing data to improve predictions. More precisely, unlabelled testing data are used during the training phase to perform an unsupervised transductive transfer learning towards the testing domain (Kong et al. 2013). This approach is particularly relevant when only a limited amount of labelled data is available while large amounts of unlabelled data can be obtained, as is the case with genetic sequence classification (Stanescu et al. 2016). Surprisingly, transductive learning has not penetrated the field of eDNA metabarcodes classification while its potential to overcome the limitations of genetic reference datasets is promising.

Using a curated genetic reference database of eDNA sequences from marine and freshwater fishes, we adapted a pre‐trained foundation model on a family‐level assignment task, using a custom classification head. To address the marked imbalance of the genetic reference database and introduce variability, we applied data augmentation in the form of single nucleotide replacement, mimicking simple synthetic mutations as a form of regularization. To simulate domain adaptive pre‐training (DAPT) in a deployment situation, we developed a transductive version of our model by conducting a pre‐training on the unlabelled test dataset. In order to assess the performance of this novel framework against tools traditionally used for eDNA assignment, we designed a comprehensive analysis comparing traditional least common ancestor (LCA) sequence alignment tools and deep learning models with our new approach on a custom dataset of short fish eDNA sequences. Finally, we assessed the performance of a hybrid transductive‐alignment framework taking advantage of both closest matches and top‐ranked families.

## 2 Materials and Methods

### 2.1 Materials

#### 2.1.1 Selection of Primers

Optimal eDNA primer choice for the purpose of biodiversity monitoring remains debated (Ruiz et al. 2025). Longer sequences tend to carry more information and lead to better taxonomic discrimination (Fontes et al. 2024), but degrade faster in a natural environment (Jo 2023), making them less detectable than shorter sequences. In order to reflect this trade‐off (precision vs. detectability) in realistic field conditions and to test taxonomic classification pipelines in suboptimal conditions on multiple sequence lengths, we selected a set of four mitochondrial primers commonly used in aquatic eDNA studies for marine and freshwater fish: Teleo (Valentini et al. 2016), MiFish‐U (Miya et al. 2015), Berry‐fish (Berry et al. 2017) and Ac16S (Evans et al. 2016) (Table 1). These primers are associated with metabarcodes that span a broad range of sequence lengths (i.e., 63 to 340 bp), allowing to test the effect of amplicon size on the performance of each model, in terms of classification accuracy. Since the considered taxa differ across primers, our selection was not intended as a direct comparison of primer performance, but rather as a way to evaluate classification pipelines across varying sequence lengths.

**TABLE 1:** Selected mitochondrial primers and representation in the final datasets.

Primer name	Region	Metabarcode length (bp)	Targeted taxa	Number of sequences	Number of families	Number of genera
Teleo	12 s	~63	Freshwater/Marine fishes	9906	306	2621
MiFish‐U	12 s	~170	Freshwater/Marine fishes	10,222	295	2479
Berry‐fish	16 s	~204	Freshwater/Marine fishes	17,863	333	3050
Ac16S	16 s	~340	Freshwater/Marine fishes	7908	258	2119

#### 2.1.2 Data Extraction

Mitochondrial sequence fragments for all markers were extracted from two databases: MIDORI2 (Leray et al. 2022) version 261 dated 2024/06/15, a curated collection of mitochondrial sequences derived from GenBank and designed for eDNA applications for 12S and 16S, and MitoFish (Zhu et al. 2023) for 12S only. For each marker, amplicon regions were extracted via in silico PCR using the crabs‐A software (Jeunen et al. 2023) on the entire mitogenome dataset, allowing a nucleotide mismatch of 3, a minimum metabarcode length of 20 for Teleo and 25 for the three other primers. Pairwise global alignment was not performed due to its addition of irrelevant sequences. We then filtered this set to retain only sequences belonging to the classes Actinopteri and Chondrichthyes, which are teleost and elasmobranch fishes.

#### 2.1.3 Data Curation and Pre‐Processing

To ensure data quality prior to training, we performed a preliminary curation of the datasets. This revealed several inconsistencies, including missing taxonomic assignments, conflicting family annotations among sequences within the same genus, and conflicting order annotations within the same family. These issues likely result from the continuous updating of the NCBI Taxonomy Database, as well as inaccuracies and inconsistencies in MIDORI and MitoFish. Taxonomic discrepancies (i.e., family and order) were manually corrected with the help of FishBase and WoRMS. Sequences containing ambiguous nucleotides were deleted. Additionally, sequence duplicates were identified and removed to limit the over‐representation of dominant taxa in the datasets. The resulting number of sequences, families and species for each primer set is presented in Table 1.

#### 2.1.4 Taxonomic Cross‐Validation and Data Splitting

To evaluate our model on unseen or unknown eDNA sequences, a taxonomy‐wise split had to be performed. Since some same‐genus species share close, if not identical, genetic sequences (Fontes et al. 2024; Polanco et al. 2021), splitting at the species level could constitute data leakage between the train and test sets and artificially inflate model performance by ignoring phylogenetic relatedness (Roberts et al. 2017). In addition, taxonomic coverage differs widely among primer sets, and whole genera missing from the reference database is a common pattern in marine eDNA studies (Fontes et al. 2024). To simulate real world conditions and mitigate the risk of data leakage, we developed a scenario where whole genera are missing from the reference database, but all families are represented. We designed a data partitioning strategy to obtain a train, validation and test split that ensured all families appeared in both the training and testing datasets. This approach also guaranteed that genera appeared either in the train or the test set with no overlap, with a partition of data close to 80% for train, 10% for validation and 10% for test.

We performed a sixfold cross‐validation to ensure that all genera appeared at least one time in the training, validation and testing datasets (Figure 1). Partitioning works as follows: for each family, if it comprised one genus, the family was removed from the dataset. If the family was composed of six or more genera, these genera were randomly split into six sets; each set was selected exactly once for the validation/test split, while the remaining five were selected for training. Within the validation/test dataset, 50% of the genera were assigned to the test set and the others to the validation set. If the validation/test part contained only one genus, it was directly assigned to the test set, and the corresponding family did not contribute to validation. For families with more than one but fewer than six genera, each of the *n* unique genera was assigned as described above, creating *n* configurations. For the remaining (6—*n*) configurations, we randomly duplicated the splits of the *n* first genera. This train/validation/test split was performed independently for each family. For each fold, genera assigned to the same set (train, validation or test) were then pooled across families to create a single train, validation and test dataset. The final dataset therefore consists of six folds, each comprising one train, validation and test set. Figure 1 illustrates an example of the procedure for three families with different numbers of genera.

**FIGURE 1:**
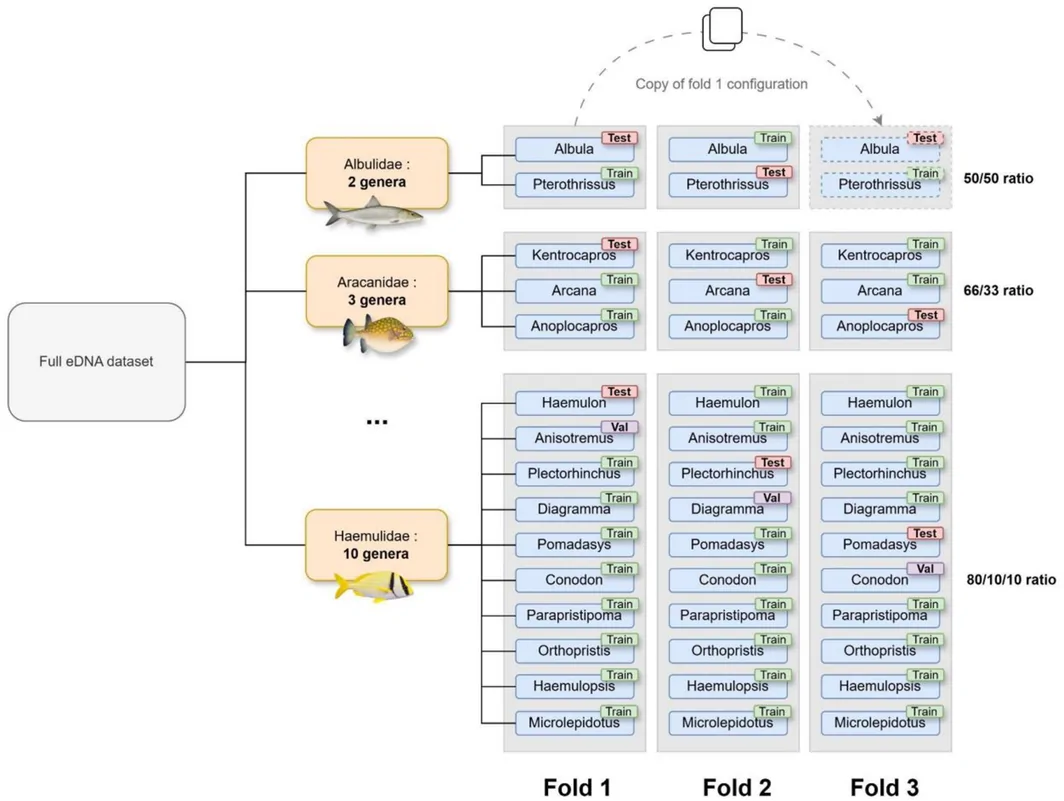
Illustration of the hierarchical split for three families represented in the teleo dataset, here shown for three distinct folds instead of six for readability. Each family is independently split so that one part of the genera is sent to the train set, and the other to the validation/test set. The split is designed to obtain an 80/20 split between train and validation/test. If there are fewer genera than there are folds (Albulidae), the maximum number of folds is reached, and the configurations are then randomly duplicated to populate the missing folds. If there are < 10 genera within a family (Albulidae and Aracanidae), the validation/test genus is automatically sent to the test set, and the family does not contribute to the validation set. If there are 10 or more genera (Haemulidae), the validation/test part is separated equally between validation and test.

#### 2.1.5 Balancing and Augmentation of the Training Datasets

To rebalance the training datasets created during the split procedure (see Section 2.1.4), we oversampled under‐represented families (size below 70% of the most represented family's sequence count) by duplicating their sequences until they reached this 70% threshold. For all markers, the most represented family was systematically the Gobiidae, with up to 511 unique sequences in a single training set for Teleo. On duplicated sequences, we injected noise through random base replacement, at a rate of 5% per base. This procedure was conducted to simulate intra‐familial diversity, thereby introducing variability in the training datasets for better generalization capabilities. This augmentation strategy is a form of regularization, which generally decreases overfitting and improves generalization performance (Wei and Zou 2019). The 5% replacement rate was experimentally determined through a step‐by‐step optimization process on all folds (Table S1), using starting rate values close to the congeneric variability of the markers used (Fontes et al. 2024).

### 2.2 Benchmarked Models

To compare the performance of our new approach to well‐established taxonomic classification methods on short and imbalanced eDNA sequences, we selected five representative approaches: Obitag from OBITools4 and MMseqs2 representing traditional LCA alignment methods, while BERTax, DNAGPT and DNABERT‐2 represent deep learning pipelines based on transformer architectures.

#### 2.2.1 OBITools4 Obitag

OBITools is a set of high‐throughput modular genomics tools tailored for the processing and analysis of eDNA metabarcoding data. It enables the treatment and filtering of raw eDNA sequences, and their taxonomic classification using local alignment against a reference database via its obitag tool. Obitag is a three‐phase process: for each test sequence, the program first searches for the most similar sequence (*Bseq*) in the reference database (primary reference). It then searches for sequences from the reference database that display a similarity ratio higher than the similarity ratio of *Bseq* with the test sequence (*Bref*). Finally, Obitag assigns the last common ancestor of all *Bref* sequences to the test sequence (Boyer et al. 2016). OBITools4 is entirely programmed in Go and open source (Metabarcoding/Obitools4 2024/2025), bringing significantly faster inference and parallelization capabilities compared to previous versions. For clarity, Obitag will be referred to as OBITools4 throughout the article.

#### 2.2.2 MMseqs2 Taxonomy

MMseqs is a high‐performance sequence alignment and clustering software suite optimized for large volumes of metagenomics data. Its taxonomy module compares query sequences against an annotated reference database and finds the LCA between matches (Steinegger and Söding 2017). MMseqs2 enables precise sequence assignment via high‐speed similarity searches and has been adopted in several eDNA annotation pipelines (Bayer et al. 2025; Patin et al. 2025).

#### 2.2.3 BERTax

BERTax is a multi‐level taxonomic classification model built on the BERT transformer architecture. It was trained and tested on microbial DNA fragments and was shown to outperform traditional alignment methods, such as MMseqs2, on organisms lacking close relatives in the training dataset (Mock et al. 2021). BERTax is used here as a state‐of‐the‐art benchmark for the deep learning classification approach of DNA sequences. It was fine‐tuned on our eDNA dataset for the family prediction.

#### 2.2.4 DNAGPT‐S‐512

DNAGPT‐S‐512 is a 0.1B parameter autoregressive transformer model based on the GPT‐2 architecture. It was trained on ~200 billion base pairs of all mammals, using three objectives: next token prediction, Guanine/Cytosine‐content regression, and sequence order prediction. The suite also includes DNAGPT‐H (human genome, 0.1B), DNAGPT‐M (9 species, 0.1B) and DNAGPT‐B‐512 (all mammals, 3B) (Zhang et al. 2023). We chose to use DNAGPT‐S‐512 due to its multi‐species and lightweight capabilities that make it comparable to our other approaches. DNAGPT‐S‐512 will be referred to as DNAGPT throughout the article.

#### 2.2.5 DNABERT‐2

Since alignment methods rely on direct comparison with reference sequences, they are relatively robust to the volume of available reference data, whereas deep learning architectures typically perform better with a large amount of training samples (Angermueller et al. 2016; Sun et al. 2017). While BERTax leverages the high availability of microbial genomic material to perform its own pretraining step on a masked language modelling (MLM) task, eDNA reference material is typically scarce for macro‐organisms, as sequences are short and taxon‐specific. This leads to comparatively small datasets and limits our capacity to effectively pre‐train our own model using eDNA amplicons. Fortunately, the recent emergence of powerful computing infrastructures has led to the multiplication of pretrained foundational models tailored for various applications. Among these, DNABERT‐2, a modified BERT model, has proved to be effective on genome classification tasks across multiple species, owing to its large, diversified pretraining dataset (Zhou et al. 2024). Thus, it constitutes a powerful and more flexible deep learning alternative to BERTax.

### 2.3 Our New *BouillaBERT* Framework

Building on DNABERT‐2, we introduce a novel transductive approach tailored to the specific task of taxonomic classification of short eDNA metabarcodes called ‘*BouillaBERT*’ (Figure 2). We named the model *BouillaBERT* after the traditional Mediterranean fish dish, as a nod to its integrative design combining information from diverse fish metabarcodes. To test the impact of both pre‐training and transductive learning, we further designed two variants: ‘BouillaBERT_rand’ for which DNABERT‐2 weights were re‐initialized, and ‘*BouillaBERT_trans’* that includes a transductive learning step before the final fine‐tuning task.

**FIGURE 2:**
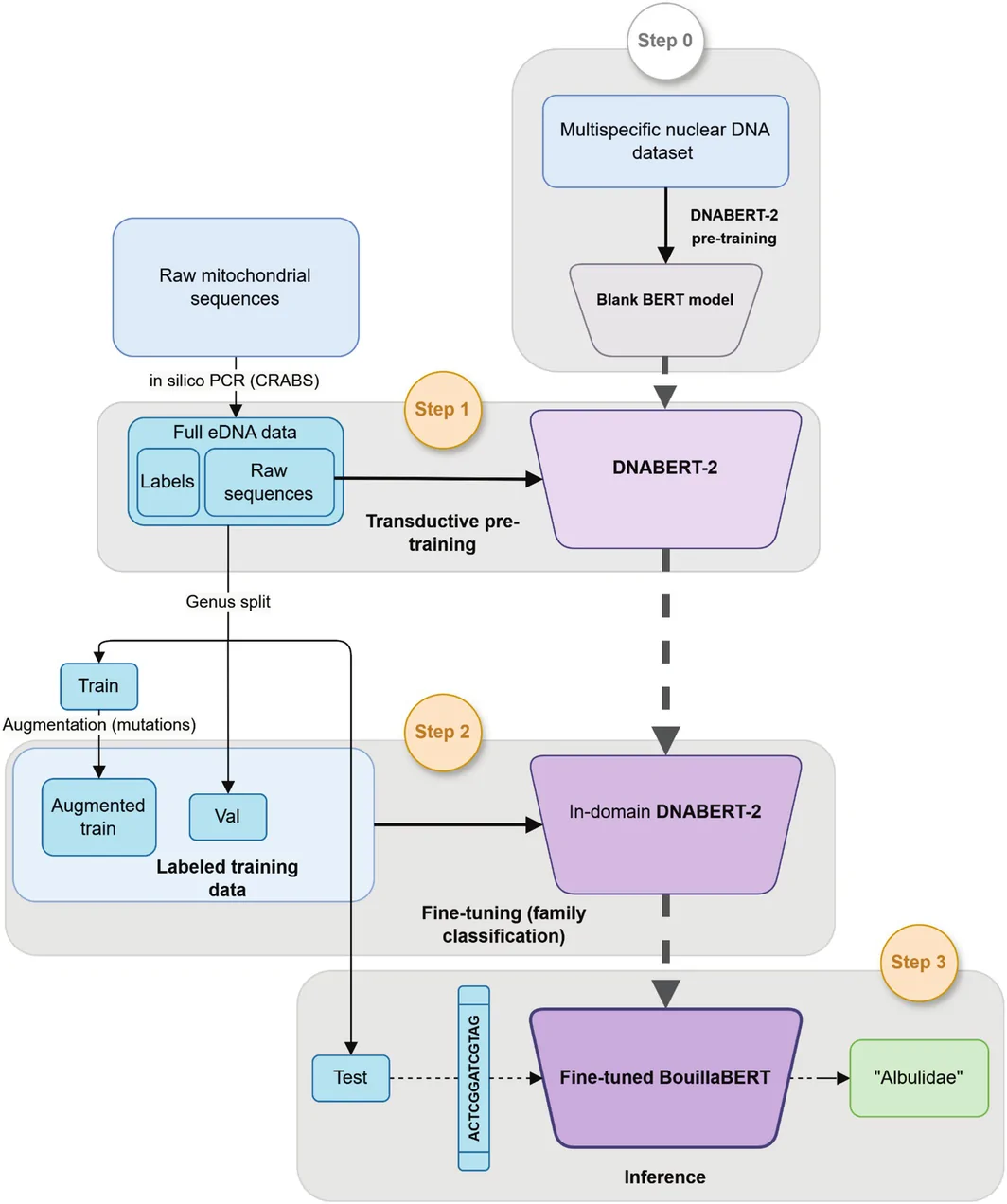
Flow chart for the transductive BouillaBERT pipeline. The workflow is based on a DNABERT‐2 foundation model, which was pre‐trained on multispecific DNA data (Step 0). The model undergoes transductive learning on the totality of the unlabelled eDNA dataset, based on a self‐supervised masked language modelling (MLM) task (Step 1). The in‐domain model is then fine‐tuned on the labelled training data (Step 2). The fine‐tuned model is used in inference to predict the family of a test sequence (Step 3). The blue boxes represent DNA data. Purple boxes represent models. The green box represents the model prediction during inference.

#### 2.3.1 Model Architecture

*BouillaBERT* is directly derived from the DNABERT‐2 encoder, which retains the standard architecture of BERT‐base, while replacing internal components to improve effectiveness, efficiency and generalization. Specifically, ‘FlashAttention’ and ‘Low Precision Layer Normalization’ provide better performance, while the implementation of ‘Attention with Linear Biases’ (ALiBi) replaces the positional embedding mechanism for better extrapolation capability. Additionally, this modified architecture uses a GeGLU activation function, a variant of GLU (Dauphin et al. 2017), which has been shown to improve the performance of transformer models on downstream tasks (Shazeer 2020). GeGLU brings the total number of parameters to 117M by enlarging intermediate layers (Zhou et al. 2024).

For our classification purposes, the encoder was fitted with a custom multi‐level taxonomic classification head. Like TaxonNet (Carranza‐Rojas et al. 2018) and BERTax, a single BERT model was used to handle multiple taxonomic levels. It first predicts a logit vector for the order from its last hidden states. A logit vector is a raw score output that represents the relative confidence for each class. It then combines this vector with the same hidden states to predict family probabilities. This favours consistency between the predicted order and family levels, allowing the model to leverage the easier‐to‐predict order probabilities to improve the accuracy of the family prediction.

#### 2.3.2 Tokenization and Input Representation

In AI and specifically transformer pipelines, tokenization is the process of partitioning data into smaller chunks called tokens, using a tool called a tokenizer. In the case of LLMs adapted to DNA data, tokens may be assimilated to k‐mers (Dotan et al. 2024). In order to use the pre‐trained weights of DNABERT‐2, the same 4096 word byte pair encoding (BPE) tokenizer was used. BPE is a tokenization method that aggregates high‐frequency character chains into tokens. This approach produces a vocabulary adapted to the training data. As a result, sequences are divided into non‐overlapping pre‐defined tokens of variable length. BPE tokenization of an eDNA sequence is represented in Figure 3.

**FIGURE 3:**
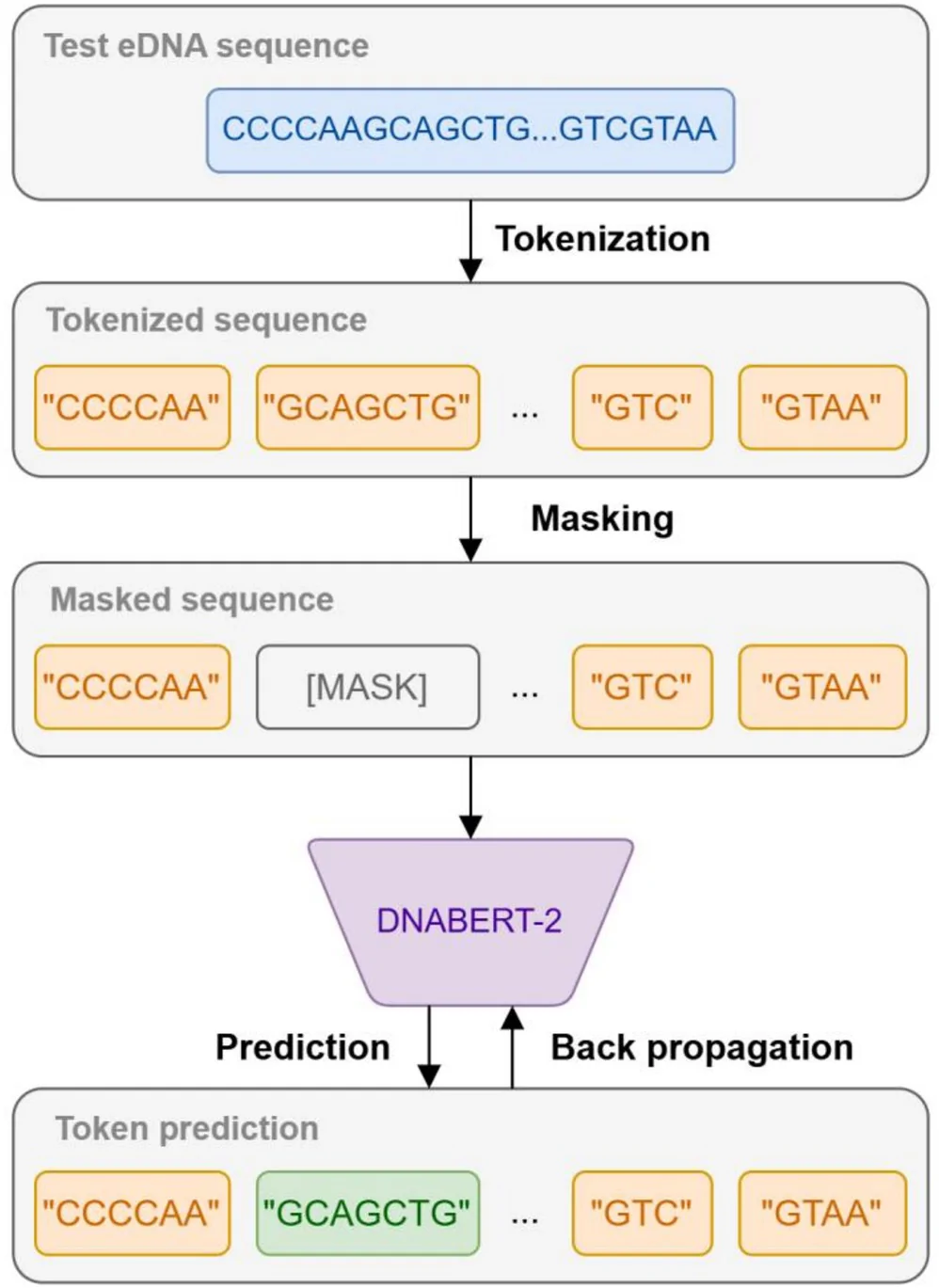
Tokenization of an eDNA sequence and masked language modelling (MLM) in a transductive learning setup. An unlabelled eDNA sequence from the test set is tokenized. A number of tokens are randomly masked and the sequence is fed into the DNABERT‐2 model. DNABERT‐2 predicts the missing token from its vocabulary, and an error value (loss) is backpropagated into the network.

#### 2.3.3 Transductive Learning (Step 1)

Before our use of DNABERT‐2, it was originally pre‐trained using a 32.49B nucleotide dataset representing the nuclear genome of 135 species spread across six categories, ranging from fungi to mammals, with a maximum sequence length of 128 tokens (Zhou et al. 2024). The use of DNABERT‐2 weights for our application thus constitutes a domain shift in origin (multispecies nuclear DNA vs. fish mitochondrial DNA), length (128 tokens vs. approximately 10 tokens) and distribution. To fill this gap and assess whether our model is able to be tailored for our specific task, we applied a second phase of DAPT, using a transductive learning approach (Step 1 in Figure 2). In transductive learning, the model undergoes additional pre‐training on the entire dataset, including the training, validation and test inputs, but only uses labels from the training set. Transductive learning aims to adapt a model to a specific target dataset using unlabelled input sequences. This situation commonly arises in eDNA workflows, where large numbers of metabarcodes are readily accessible prior to assignment. The model can therefore exploit information contained in the sequence composition of the target dataset while remaining agnostic to the taxonomic labels used for evaluation. This is a natural capability of deep learning approaches, especially large language models (LLM) (Gururangan et al. 2020). For this step, *BouillaBERT* was trained using MLM with a masking proportion of 20% on the whole eDNA dataset (Figure 3). Transductive pre‐training hyperparameters are presented in Table S2. This variant of our model is called *BouillaBERT_trans*.

#### 2.3.4 Supervised Fine‐Tuning (Step 2)

We then fine‐tuned the pretrained model (either *BouillaBERT_rand*, *BouillaBERT* or *BouillaBERT_trans*) on the final supervised classification tasks (at the *order* and *family* levels) using our custom hierarchical classification head (Step 2 in Figure 2). All weights were unfrozen for this step. Both stages of the hierarchical head (final layer) were trained jointly using a cross‐entropy loss function for each of them. Contrary to Step 1 (transductive learning), only the samples of the training set were used for training while the held‐out validation set was used for model selection. Loss values and classification metrics were computed for the validation set every 1200 steps. Weights were saved regularly, and the best model was selected based on the macro‐averaged validation f1 score for family classification. Macro‐averaged validation f1 score represents the ability of the model to balance precision (avoiding false positives) and recall (avoiding false negatives) for the validation set, averaged across all classes. It constitutes a good indicator of its general performance.

#### 2.3.5 Random Initialization Test

To assess the impact of DNABERT‐2 pre‐training (Step 0) on *BouillaBERT*'s performance, we conducted a random initialization test by resetting the weights of the pretrained model and repeating the fine‐tuning step. This model is called *BouillaBERT_rand*.

#### 2.3.6 Hybrid Top‐15 Pipeline

We finally developed a hybrid pipeline to combine the strength of alignment‐based and deep learning approaches. For each test set, we checked whether OBITools4 and MMseqs2 predicted fish families appeared in the corresponding transductive *BouillaBERT_trans* top‐15 predictions; if not, we replaced them with *BouillaBERT_trans* top predictions to avoid misclassification by alignment‐based methods.

### 2.4 Experimental Design

#### 2.4.1 Comparison of Approaches

In order to compare the performance of our method, we used the same datasets to train and run OBITools4 obitag, MMseqs2 taxonomy, BERTax, DNAGPT, *BouillaBERT* and Hybrid Top‐15 for each marker, and all models were evaluated on the same test sets. For OBITools4 and MMseqs2, the train sets were used as reference databases. This setup reflects our objective of simulating assignment with incomplete reference databases, where entire genera are absent from the available reference sequence collection. OBITools4's assignments were kept when their predicted LCA were at or below the family level, while MMseqs2 was run with parameters optimized on the validation set. The parameters for MMseqs2 are available in the Note S1. BERTax, DNAGPT and *BouillaBERT* were fine‐tuned on the train sets and evaluated using their top‐1 predictions.

#### 2.4.2 Metrics and Statistical Tests

Since our datasets are highly imbalanced, we reported macro‐averaged metrics to provide equal weight to all families, regardless of their representation. Macro‐averaged precision represents the proportion of predicted fish families that are correct, penalizing false positives. In an ecological context, low precision would mean that the algorithm falsely predicts taxa that are absent. Macro‐averaged recall represents the proportion of families that are correctly identified (true positives), penalizing false negatives. In an ecological context, low recall would mean that the algorithm fails to detect taxa that are present. Finally, the macro‐averaged F1‐Score was used to assess the balance between precision and recall. We measured statistical significance of the differences in performance using an exact paired Wilcoxon signed‐rank test, since our number of samples is small (sixfold).

#### 2.4.3 Fold‐Averaged Recovery Rate

For each family and alignment method, we computed the fraction of cases where the alignment method predicted incorrectly but *BouillaBERT* predicted correctly. Percentages were computed per fold and averaged across folds. It is referred to as the fold‐averaged recovery rate.

## 3 Results

### 3.1 Comparison of *BouillaBERT* Variants

We first assessed the effect of pretraining and transductive learning on *BouillaBERT* performance (macro‐averaged P/R/F1, six folds) by comparing the randomized *BouillaBERT* (*BouillaBERT_rand*), the pre‐trained inductive *BouillaBERT* (*BouillaBERT*) and the transductive *BouillaBERT* (*BouillaBERT_trans*).

Across all markers, the pretrained *BouillaBERT* consistently outperformed the untrained (randomized) version (Figure 4, Table 2). In macro‐averaged precision, so the capacity to ensure correct family‐level assignments (true positives), pretraining improved predictions by +9.5/+4.0/+5.9/+1.1 points for Teleo/MiFish/Berry/Ac16S markers, respectively, with a significant gain for teleo, MiFish and Berry (*p* = 0.031) and non‐significant for Ac16S (*p* = 0.219). For macro‐averaged recall, so the ability to avoid missing identifications across families (false negatives), the pretrained *BouillaBERT* outperformed the random *BouillaBERT* by +9.0/+3.5/+3.9/+0.7 points for teleo/MiFish/Berry/Ac16S, respectively, with the difference being significant for all markers (*p* = 0.031) but Ac16S (*p* = 0.438). Finally, in macro‐averaged F1, which indicates the balance between precision and recall, pretraining improved fish family predictions by +9.3/+4.0/+5.1/+1.2 points for teleo/MiFish/Berry/Ac16S, respectively, compared to a random version. These gains were significant for teleo and Berry (*p* = 0.031), marginally significant for MiFish (*p* = 0.062), but non‐significant for Ac16S (*p* = 0.312). Overall, the predictive gain of pre‐training *BouillaBERT* was particularly marked for the shortest marker (Teleo) and marginal for the longest marker (Ac16S).

**FIGURE 4:**
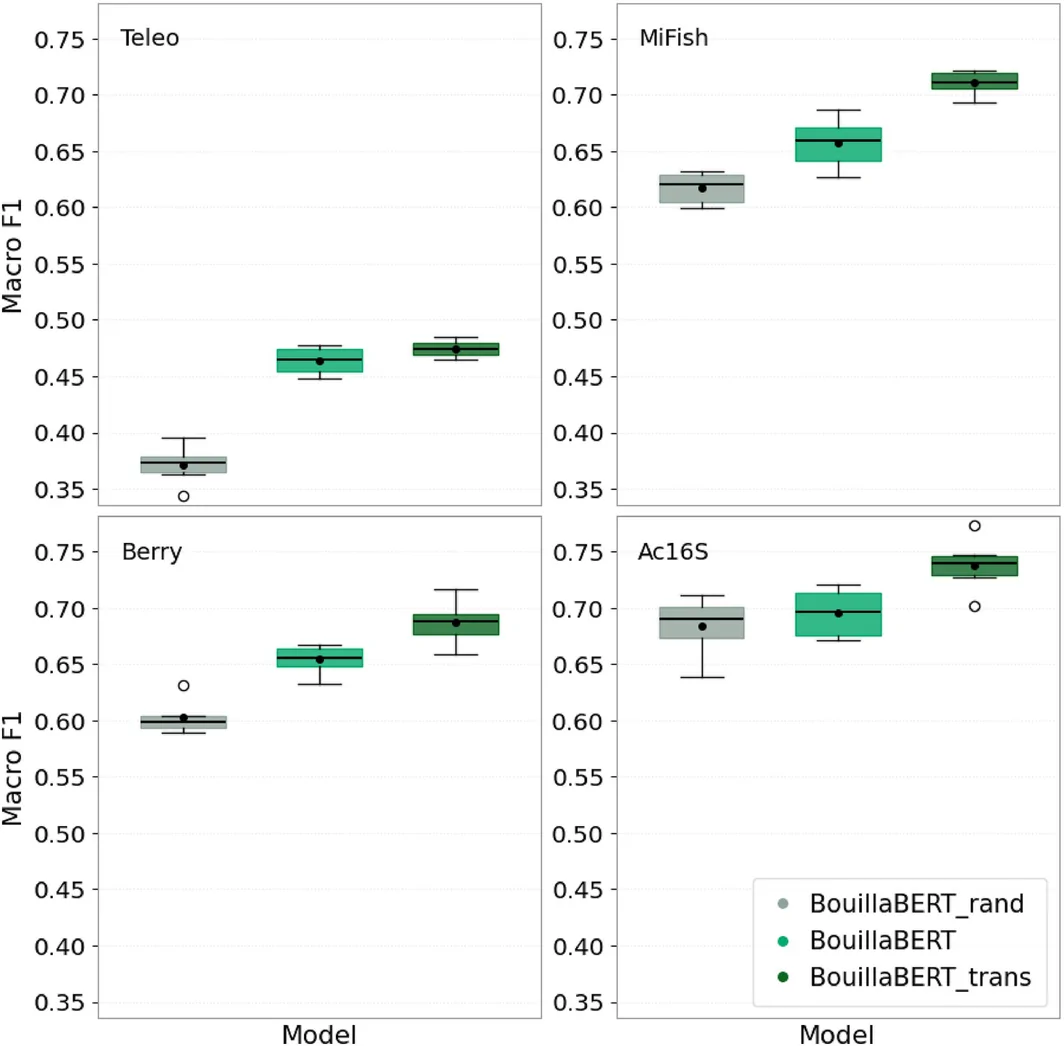
Performance comparison of BouillaBERT variants (BouillaBERT_rand is the randomized version, BouillaBERT is the standard inductive version, BouillaBERT_trans is the transductive version) across four markers (Teleo, MiFish, Berry, Ac16S). Results are shown as macro‐averaged F1‐score over six cross‐validation folds. Boxplots summarize the distribution of fold‐wise performance (median, interquartile range, whiskers extending to 1.5 × IQR, with outliers shown as open circles). Black dots indicate the mean Macro F1 across folds.

**TABLE 2:** Mean macro averaged precision (Prec), recall (Rec) and F1‐score (F1) (sixfold cross validation), computed on the test sets, on all markers and for all approaches. *BouillaBERT_rand* is the randomized version of our model. *BouillaBERT_trans* is the transductive version. Hyb_obi4 and Hyb_mmseqs2 are the hybrid approaches using, respectively, OBITools4 and MMseqs2. Scores are averaged across all six folds.

		Teleo			MiFish			Berry			Ac16S		
		Prec	Rec	F1	Prec	Rec	F1	Prec	Rec	F1	Prec	Rec	F1
Alignment	OBITools4	**0.456**	0.407	0.413	0.639	0.602	0.605	**0.674**	0.621	0.628	0.634	0.595	0.600
	MMseqs2	0.430	0.483	0.433	0.620	0.684	0.627	0.639	0.696	0.637	0.659	0.725	0.670
Deep learning	Bertax	0.306	0.387	0.313	0.471	0.557	0.480	0.456	0.542	0.457	0.496	0.582	0.508
	DNAGPT	0.412	0.491	0.420	0.564	0.637	0.574	0.626	0.697	0.628	0.620	0.693	0.632
	BouillaBERT_rand	0.354	0.451	0.371	0.606	0.686	0.617	0.591	0.687	0.603	0.669	0.747	0.684
	BouillaBERT	0.449	**0.540**	**0.464**	**0.647**	**0.721**	**0.657**	0.650	**0.726**	**0.654**	**0.679**	**0.754**	**0.696**
	BouillaBERT_trans	**0.459**	**0.551**	**0.474**	**0.698**	**0.768**	**0.710**	**0.680**	**0.758**	**0.687**	**0.724**	**0.786**	**0.738**
Hybrid	Hyb_obi4	**0.475**	**0.568**	**0.491**	**0.701**	**0.773**	**0.714**	**0.696**	**0.772**	**0.714**	**0.740**	**0.802**	**0.755**
	Hyb_mmseqs2	0.462	0.556	0.479	0.678	0.751	0.691	0.673	0.746	0.678	0.696	0.763	0.710

*Note:* Bold values indicate the best‐performing model within each class (non‐hybrid and hybrid methods); they do not indicate statistical significance. For non‐hybrid methods, both BouillaBERT and BouillaBERT_trans were bolded when their scores were higher than those of the baseline methods; otherwise, the best‐performing baseline was bolded.

Across all markers, the *BouillaBERT_trans* version consistently outperformed the standard pre‐trained version. It improved the macro‐averaged precision by +1.0/+5.1/+3.0/+4.4 points for Teleo/MiFish/Berry/Ac16S markers, respectively. These differences were statistically significant for all markers (*p* = 0.031). In macro‐averaged recall, *BouillaBERT_trans* also outperformed the standard *BouillaBERT* by +1.0/+4.7/+3.1/+3.2 points for Teleo/MiFish/Berry/Ac16S markers, respectively, with all differences being significant (*p* = 0.031). Finally, the transductive approach improved macro‐averaged F1 by +1.0/+5.3/+3.3/+4.2 points for Teleo/MiFish/Berry/Ac16S markers, respectively, compared to the standard *BouillaBERT*. The gain was significant for MiFish, Berry and Ac16S (*p* = 0.031) and marginally significant for Teleo (*p* = 0.062).

### 3.2 *BouillaBERT* Versus Alternative Approaches

To assess how well *BouillaBERT* performs against state‐of‐the‐art methods, we then compared its best version (*BouillaBERT_trans*) against alignment (OBITools4, MMseqs2) and deep learning baselines (BERTax, DNAGPT).

Across all markers, *BouillaBERT_trans* consistently outperformed all other approaches in both macro‐F1 and macro‐recall (Figure 5, Table 2). In macro‐precision, *BouillaBERT_trans* outperformed OBITools, the best external baseline by +0.3/+5.8/+0.5 points for Teleo/MiFish/Berry, respectively, and MMseqs2, the best external baseline for Ac16S, by +6.5 points. This difference is significant for MiFish and Ac16 (*p* = 0.031) and not significant for Teleo and Berry (*p* = 0.562 and 0.688 respectively). For macro‐recall, the best comparative baseline varies among markers with DNAGPT for Teleo and Berry and MMseqs2 for MiFish and Ac16S, but is systematically and significantly (*p* = 0.031) outperformed by the *BouillaBERT_trans* with increases of +5.9/+8.4/+6.1/+6.1 points for Teleo/MiFish/Berry/Ac16S, respectively. In macro F1, the gain provided by *BouillaBERT_trans* over the best baseline, MMseqs2, reached +4.2/+8.4/+5.0/+6.8 points for Teleo/MiFish/Berry/Ac16S, respectively, with the Wilcoxon exact test being significant for all markers (*p* = 0.031). Despite its relatively good precision, OBITools assigned a high number of taxa as unknown (NC): on average across folds, 46.76%/28.75%/29.23%/31.78% of all unknown sequences for Teleo/MiFish/Berry/Ac16S markers, respectively, are classified as ‘NC’. Comparatively, *BouillaBERT_trans* is much less conservative since it always provides a prediction.

**FIGURE 5:**
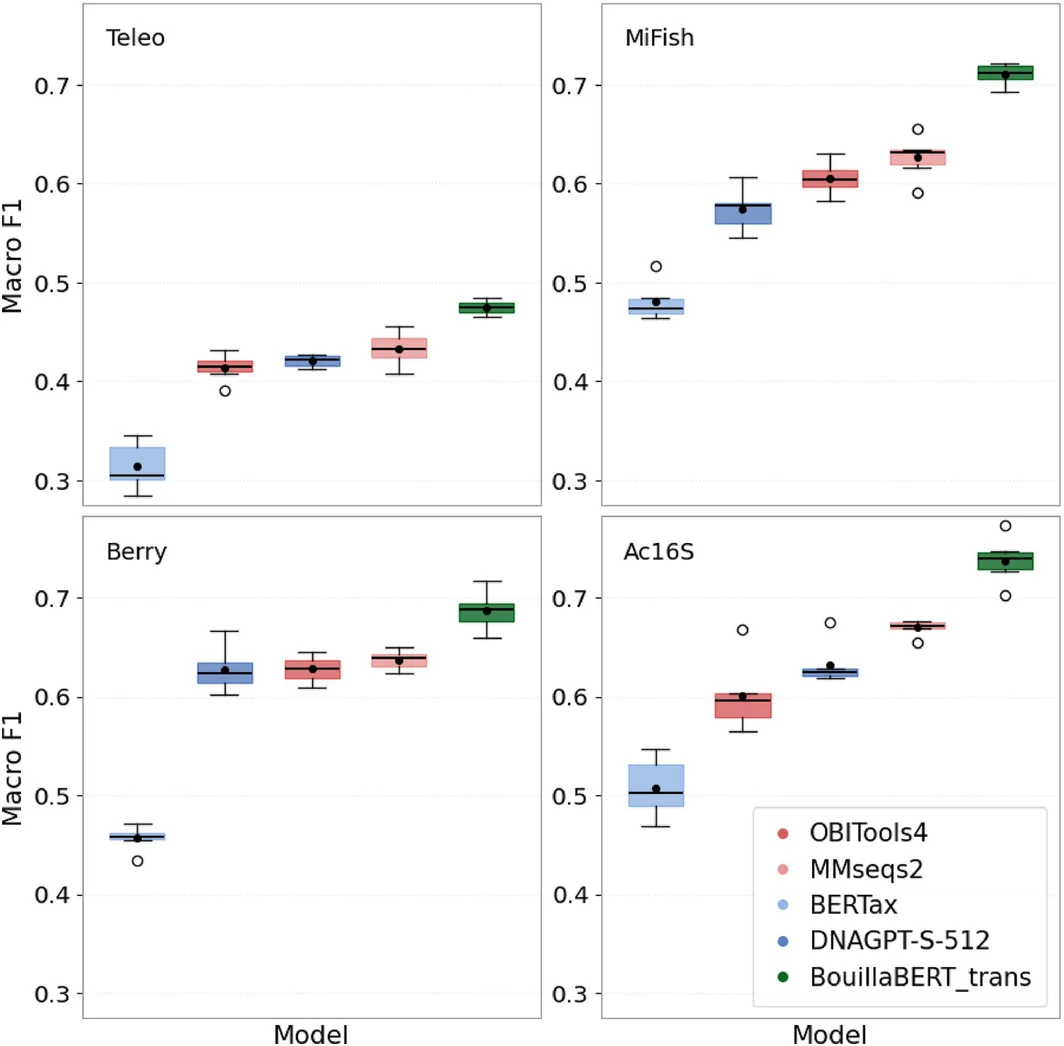
Performance comparison of BouillaBERT_trans with external baselines across four markers (Teleo, MiFish, Berry, Ac16S). Results are shown as macro‐averaged F1‐score over six cross‐validation folds. Boxplots summarize the distribution of fold‐wise performance (median, interquartile range, whiskers extending to 1.5 × IQR, with outliers shown as open circles). Black dots indicate the mean Macro F1 across folds. Methods are ranked by increasing performance but keep the same colours across panels with alignment‐based in red and AI‐based in blue, while the new BouillaBERT_trans framework is in green.

### 3.3 Hybrid Approach

OBITools4, MMseqs2 and *BouillaBERT* tend to follow similar family‐level patterns (Figure 6; *p* < 0.05), yet their relative performance is highly variable, with *BouillaBERT* outperforming MMseqs2 and OBITools4 across numerous families, while underperforming for others. At the family level, we observed this complementarity through the analysis of recovered cases. In particular, *BouillaBERT* corrected a large fraction of alignment misidentifications in some important taxonomic groups. For Siluridae, the fold‐averaged recovery rate was 74.4% for OBITools4 and 66.0% for MMseqs2 (pooled counts across folds 27/31, 11/14). For Albulidae, recovery rates reached 54.1% and 41.0% (25/45, 16/36), and for Istiophoridae, 77.8% and 17.5% (10/12, 9/11) for Obitag and MMseqs2, respectively. Detailed per‐family classification metrics for each marker and classification method are provided in Data S1.

**FIGURE 6:**
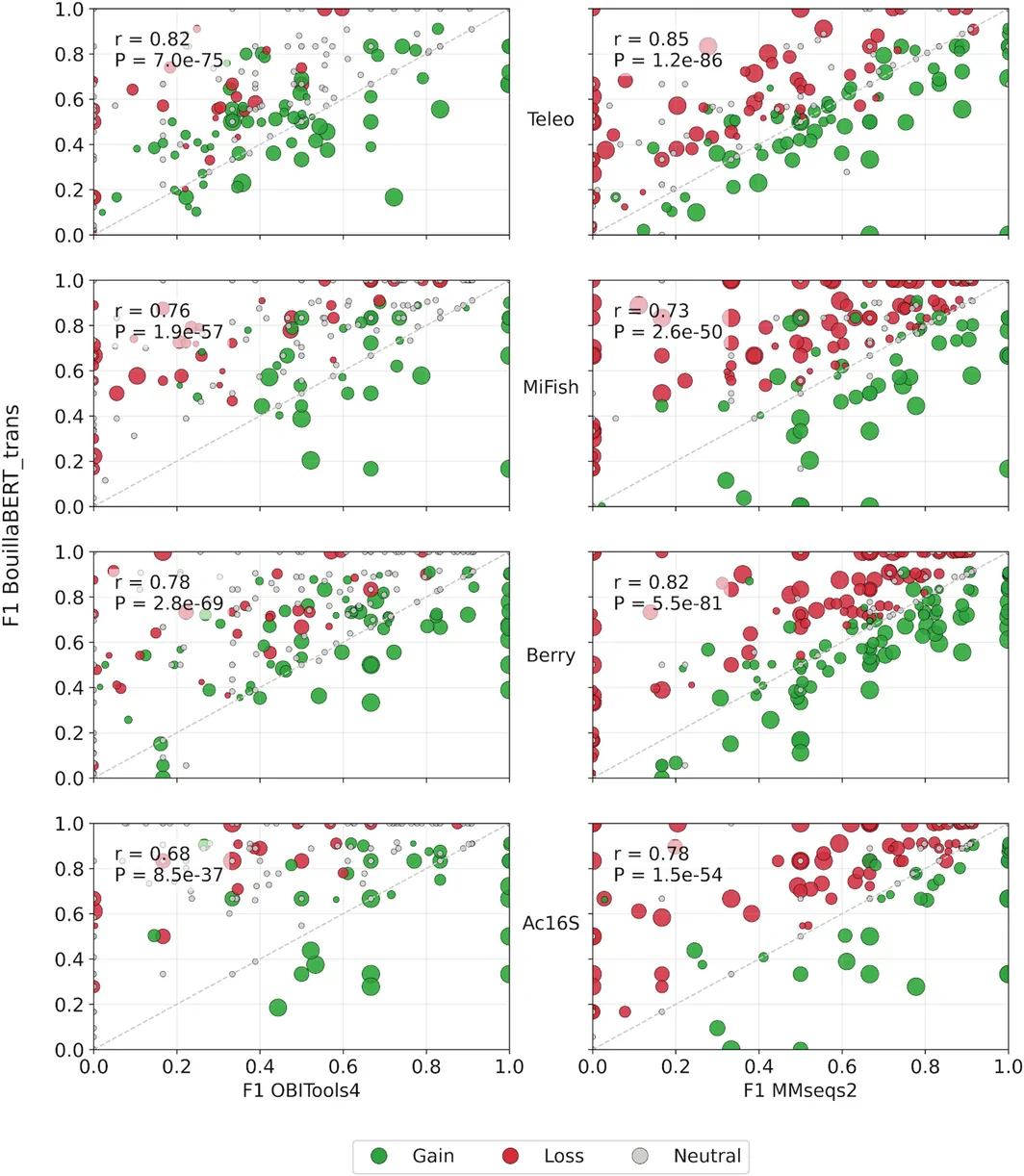
Comparison of F1‐scores between transductive BouillaBERT and OBITools4 or MMseqs2 across all predicted families, shown separately for each marker. Each dot corresponds to a family. Pearson's correlation coefficients (*r*) and associated *p*‐values (*p*) are reported. The green dots indicate families that gain from hybridization, while the red dots indicate families that lose. Dot size reflects the magnitude of the gain or loss. Families above the diagonal are better predicted by BouillaBERT_trans than by the corresponding alignment method, whereas families below the diagonal are better predicted by the alignment method.

To quantify this complementarity, we compared the performance of both hybrid approaches against their individual components. We observed that both hybrid methods generally outperformed all other pipelines (Figure 7). Specifically, the BouillaBERT‐OBITools4 hybrid approach takes the lead over the *BouillaBERT_trans* model in macro‐F1 by +1.7/+0.4/+2.7/+1.7 points (*p* = 0.031/0.219/0.031/0.031), in macro‐recall by +1.7/+0.5/+1.4/+1.6 points (*p* = 0.031/0.156/0.062/0.031), and in macro‐precision by +1.6/+0.3/+1.6/+1.6 points (*p* = 0.031/0.219/0.031/0.031). In terms of F1 performance, hybridization benefited both OBITools4 and MMseqs2, with 17.7% and 22.4% of families showing gains (averaged across all markers), respectively. At the same time, 9.7% of families experienced a performance decline when using the OBITools4 hybrid, compared with a much larger decline of 28.0% with the MMseqs2 hybrid. While the gains observed with the OBITools4 hybrid were distributed more evenly across families that were already better predicted by both *BouillaBERT_trans* and OBITools, the gains with the MMseqs2 hybrid were more narrowly concentrated among families for which MMseqs2 alone provided stronger predictions (Figure 6).

**FIGURE 7:**
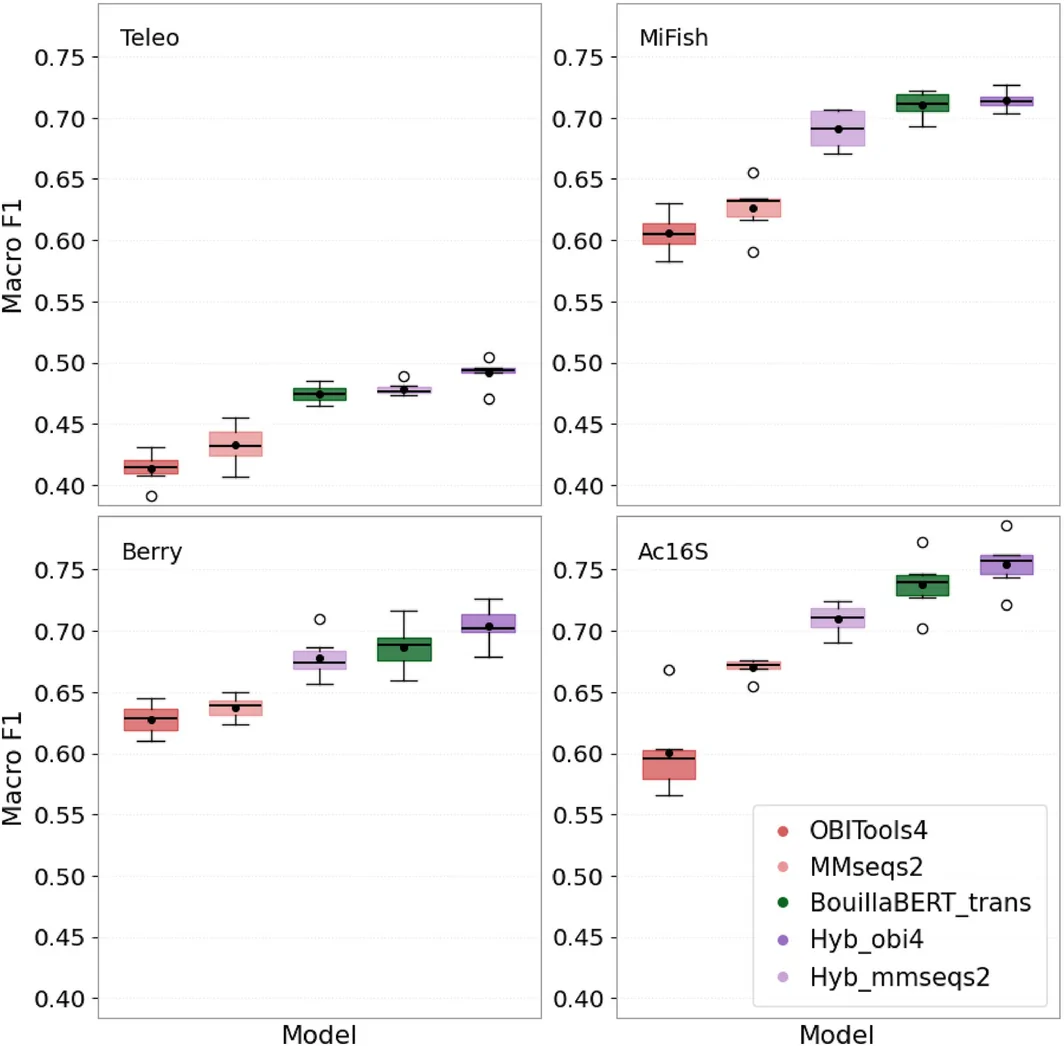
Performance comparison of hybrid approaches with their individual components across four markers (Teleo, MiFish, Berry, Ac16S). Results are shown as macro‐averaged F1‐score over six cross‐validation folds. Boxplots summarize the distribution of fold‐wise performance (median, interquartile range, whiskers extending to 1.5 × IQR, with outliers shown as open circles). Methods are ranked by increasing performance but keep the same colours across panels with alignment‐based in red and AI‐based in blue, while the new BouillaBERT_trans framework is in green.

## 4 Discussion

### 4.1 Fine‐Tuning a Foundation Model Improves Predictions

Our results first demonstrate that a pre‐trained foundation model generally outperforms baseline methods, including both alignment (OBITools4, MMseqs2) and deep learning (BERTax, DNAGPT) approaches, when classifying eDNA sequences belonging to genera excluded from training, a scenario that mimics incomplete reference databases. The superiority of our new *BouillaBERT* framework over LCA alignment models, specifically for unknown genera, is in line with the results of BERTax (Mock et al. 2021). However, the advantage of *BouillaBERT* over BERTax, as well as the major gain of *BouillaBERT* over its randomized version, shows that sharing the same classification task is not sufficient: fine‐tuning a generalist foundation model such as DNABERT‐2 proves more effective than adapting a same‐purpose model trained on unrelated and too specific data, such as BERTax. Reaching a balance between the flexibility of the pre‐trained model and its specificity to the target data therefore appears essential for building accurate assignment methods for eDNA pipelines (Han et al. 2021). Pre‐training seems especially effective on short markers like Teleo, with a performance gain considerably higher than with Ac16S, the longest marker in the set. This suggests that knowledge contained in the pre‐trained weights becomes increasingly relevant when samples lack usable features like metabarcode information. However, the split procedure relies on fixed rules, while reference database overlap across markers remains only partial. As a result, each marker yields a different taxonomic composition and data split. Some species represented for one marker are absent for another, making the benchmark unsuitable for isolating the effect of amplicon length on classification performance. Such an analysis would require restricting all reference databases to the taxa shared across all markers.

These results imply that pre‐trained foundation models can act as an external source of knowledge that compensates for the lack of taxonomic information contained in short markers, which are most often used. This is an essential aspect in the field of eDNA metabarcoding due to fast DNA degradation in the environment (Jo 2023). Similar strategies could be extended to other widely used markers such as 98‐nucleotides‐long 12S‐V5 for vertebrates (Riaz et al. 2011), suggesting that the fine‐tuning of foundation models has a broad potential across biodiversity monitoring applications.

Interestingly, our randomized version of *BouillaBERT* still performs better than BERTax, especially on longer markers like Ac16S. The main difference between the models is the enlarged intermediate layer *BouillaBERT* inherits from DNABERT‐2, caused by the use of the GeGLU activation function, suggesting that the classification performance is a function of the size of the model. This result is in line with previous work on DNA foundation models (Dalla‐Torre et al. 2025). In future research and with the development of more powerful consumer‐grade systems, we may expect larger language models to be used for such classification tasks with little additional training time, leading to increased performance. All experiments were carried out on a single DELL Precision 3660 work station, using an NVIDIA RTX A6000. Each full training run, including both further pre‐training and fine‐tuning, was completed in approximately 5 h. This relatively modest computational demand qualifies *BouillaBERT* for use in a biodiversity monitoring context without the need for specialized computing clusters.

### 4.2 A Transductive Approach Boosts *BouillaBERT* Performance

We find that our transductive *BouillaBERT* model performs the best on all metrics and for all markers. Although pretraining a model on the unlabelled test set may seem counterintuitive, doing so enables the model to learn the distribution and the genetic specificities of the test samples. This approach relies on the assumption that unlabelled datasets are generally available in a field setting—in our case, unknown eDNA sequences—and may be leveraged to strengthen a model's performance (Ouchi et al. 2019). Traditional approaches do indeed lack the ability to do so, which represents a missed opportunity in an already data‐poor context. Deep learning models are inherently capable of leveraging unlabelled samples; therefore, it seems relevant to exploit such available information in an operational goal such as taxonomic assignments of unknown eDNA sequences. Additionally, the relatively small size of the BERT model, combined with the use of pre‐trained weights, allows for fast implementations with new datasets, especially in a deployment context. In practice, it would require a periodical re‐training of the model, including both the transductive and fine tuning steps, on new eDNA data. Depending on the freshness of this training, real performances would fall between those of *BouillaBERT* and *BouillaBERT_trans*, which are in either way at least on par with baseline approaches. In the future, semi‐supervised or continual learning strategies could extend this idea even further, allowing models to adapt rapidly as new eDNA samples accumulate, labelled or not. However, we must acknowledge that the transductive learning step is adapted to situations where the target test dataset represents a substantial part of the total data available. So, scenarios where few unknown sequences would have to be assigned would likely not benefit much from transductive learning.

### 4.3 Comparable Precision but Better Recall Make *BouillaBERT* a Promising Tool for Conservation

We observe comparable or better precision of *BouillaBERT* over OBITools4, showing that both methods are similar in reliability for assigned sequences. OBITools notably reaches this precision by being more conservative, with a high level of uncertain predictions (28.75% to 48.76% depending on the marker) reducing the number of errors. This allows OBITools4 to provide more precise but incomplete inventories, potentially undermining conservation efforts by excluding some sequences from the final classification results, particularly those corresponding to the most vulnerable taxa like sharks (Pichot et al. 2025). More generally, missed detections of invasive species or endangered taxa could have disproportionate consequences for management decisions (Gu and Swihart 2004).

Our standard *BouillaBERT* and its transductive version are systematically higher in recall than all other baselines with average differences of approximately +3.6/+6.65 points over best‐performing baseline (standard and transductive version, respectively, averaged across markers), meaning that it is less likely to miss families of unknown genera. This may be advantageous in conservation contexts where false negatives are considered more detrimental than false positives and where positive detections can be verified through complementary surveys or observations. This aligns with the precautionary principle in biodiversity monitoring, where failing to detect a species that is present can be more harmful than mistakenly reporting a species that is absent (Bennett et al. 2024; Tyre et al. 2003).

Nevertheless, the consequences of false positives must not be underestimated. In the case of biodiversity inventories, false positives may lead to biased estimates of habitat occupancy and species distribution. As a result, conservation decisions may become misdirected, with potential effects often observed only decades later (Loiselle et al. 2003). Consequently, false positives can erode the trust of stakeholders in eDNA inventories and lead to resource waste and ineffective policies. As *BouillaBERT* provides comparable or better precision than the baselines (on average: −0.7/+3.3 points over best baseline across markers), it represents a good compromise between false negatives and false positives. The transductive version is especially effective at mitigating false positives while simultaneously decreasing false negatives.

Three families illustrate that misclassification is an ecological issue rather than a purely statistical one and that it can often be corrected by our approach. Albulidae (bonefish) include many data deficient and vulnerable species. The majority of vulnerable species from this group are located in the Coral Triangle (Adams et al. 2014), where the eDNA reference database remains notably incomplete (Juhel et al. 2020). Istiophoridae (billfish) are apex predators with keystone roles. Their ecological and economic importance requires precise knowledge of their preferred habitats (Kitchell et al. 2006; Uozumi 2003). Siluridae (freshwater catfish) include many endemic and endangered species (Haryono et al. 2024). For these three families, *BouillaBERT*'s average recovery rate ranged from 41.0% to 77.8% of the test samples that had been misidentified by both alignment methods. This highlights the potential of our method to offer a safeguard against missing such sensitive taxa.

Yet, conservation objectives are numerous, and their sensitivity to both false negative and false positive rates can vary. Consequently, the relative suitability of alignment‐based and deep learning approaches is likely to depend on the context and objectives and should be further assessed using real eDNA data.

### 4.4 Hybrid Approaches as the Best of Two Worlds

Our results demonstrate that hybrid approaches exploit the family‐level performance complementarity between baseline models and *BouillaBERT*. Our BouillaBERT‐OBITools4 hybrid provides the best performance of all methods, across all markers. This highlights a synergy between both approaches, combining the high precision of OBITools on non‐‘NC’ cases (few false positives), and the high recall of *BouillaBERT* which can recover a broader range of sequences (few false negatives). The distribution of gains and losses further supports this interpretation: improvements with the OBITools4 hybrid model are spread more homogeneously across families, consistent with a complementary relationship between the two methods, whereas MMseqs2 gains are concentrated among families it already predicts well, suggesting reinforcement rather than complementarity. Additionally, the high rate of losses experienced by MMseqs2 through hybridization explains the poor performance of the BouillaBERT_trans/MMseqs2 hybrid. This result is in line with BERTax (Mock et al. 2021), although our method allows for more flexibility while avoiding the limitations imposed by the alignment's NC assignments. This strategy may become more useful with the use of OBITools raw outputs, which could then be filtered by *BouillaBERT*'s top‐15 without limitations. In the case of MMseqs2, it would allow one to exploit alignment with low stringence, while safeguarding it from outputting anomalous assignments. The stringence parameters of the alignment tools should be further optimized for use in a hybrid pipeline.

## 5 Conclusions

Implementing a deep learning pipeline in the context of eDNA assignment is a challenging task: data are scarce, sequences are short and lack taxonomic resolution, training datasets are extremely imbalanced, and often contain annotation errors. This study demonstrates that while such a context poses a considerable challenge, using a combination of manual curation, informed data augmentation and domain‐adaptive pre‐training of foundation models helps overcome these difficulties and achieve accurate eDNA assignments in data‐poor conditions. This work suggests that foundation model‐based pipelines represent a promising path to monitor biodiversity via eDNA inventories, despite incomplete reference databases. At the same time, they should be viewed as complementary to ongoing efforts aimed at expanding and improving reference databases, not as a replacement for them.

Nevertheless, this study also has its limitations: first, only four eDNA markers have been tested, which does not cover the entire field of possible eDNA workflows, especially given the amount of markers tailored for specific case‐uses and groups (Othman et al. 2021; Ruiz et al. 2025). Among these, we manually curated datasets, but errors may remain, which would be detrimental to both traditional and novel methods (Keck et al. 2023). Finally, rare families are frequently poorly predicted, which highlights the need to sample more individuals in order to enrich reference datasets in data‐poor areas and for unrepresented taxonomic groups (Juhel et al. 2020; Sanchez et al. 2025). Another promising path would be to incorporate phylogenetic information or environmental metadata, which could guide assignments for poorly represented taxa (Polanco et al. 2025).

Our findings suggest that deep‐learning pipelines can effectively be used in the context of biodiversity monitoring for data‐poor environments, either in hybrid workflows together with alignment tools or as standalone approaches. We recommend the use of standard alignment methods in cases where reference databases are near complete and few sequences are unsuccessfully assigned, like in the Mediterranean Sea (Pichot et al. 2025), but advise the implementation of deep learning whenever taxa are not referenced and missing annotations could become detrimental to decision making (hardly accessible environments, endangered taxa, invasive or keystone species etc.). We further suggest that conservation actors willing to adapt these tools to their settings should consider the deployment of transductive methods in general and specifically in the context of language‐model based classifiers such as *BouillaBERT*, since they are fast to implement while providing significant performance improvements over standard versions.

## Author Contributions

Simon Bettinger, Auguste Verdier and David Mouillot conceived the ideas and designed the methodology, with support from all co‐authors. Auguste Verdier and Simon Bettinger collected and pre‐processed the data, trained the deep learning models, and analysed the results. Auguste Verdier and Morgane Bruno ran the alignment models. Simon Bettinger led the writing of the manuscript. All authors contributed critically to the drafts.

## Funding

Simon Bettinger's PhD scholarship was funded by the PPR Océan & Climat programme. This research was supported by the BPI project SEAMAP, the IA‐Biodiv ANR project Fish‐Predict (ANR‐ 21‐ AAFI‐ 0001‐ 01) and the ANR Chaire Industrielle ADNeIA (ANR‐25‐CHIN‐0007).

## Disclosure

Benefit Sharing: This study uses only publicly available genetic reference sequences from MIDORI2 and MitoFish. No new genetic resources were sampled or accessed.

## Ethics Statement

This study used only publicly available genetic reference sequences from MIDORI2 and MitoFish. No experiments involving humans or animals were performed, and ethical approval was not required.

## Conflicts of Interest

The authors declare no conflicts of interest.

## Supporting information


**Data S1:** Per‐family classification performance. CSV file containing classification metrics at the family level for each metabarcoding marker and classification method.

**Table S1:** Macroaveraged F1 scores for the augmentation optimization tests. Each value is an average of scores across all folds for each marker.
**Table S2:** Training hyperparameters for the transductive pre‐training and supervised fine‐tuning phases.
**Note S1** MMseqs2 command line.
**Figure S1:** Macro averaged recall, precision and F1 optimization curves for Mmseqs2, across all markers.

## Data Availability

Data and code available from Zenodo (https://doi.org/10.5281/zenodo.22204444).
