# Supplementary material for: Transductive Learning From a Transformer Foundation Model Improves the Taxonomic Classification of eDNA Metabarcodes
Source: Mol Ecol Resour. 2026 Nov 11;26(8):e70204. doi: 10.1111/1755-0998.70204 (PMC13754536; doi:10.1111/1755-0998.70204)
Supplement: Supplementary file 1 [file MEN-26-e70204-s001.docx]

# SUPPLEMENTARY MATERIALS

This Supplementary Material accompanies the manuscript **Transductive learning from a transformer foundation model improves the taxonomic classification of eDNA metabarcodes**.

Table S1 : Macroaveraged F1 scores for the augmentation optimization tests. Each value is an average of scores across all folds for each marker.

| Marker name | 2% rate | 5% rate | 10% rate | 15% rate |
| --- | --- | --- | --- | --- |
| Teleo | 0.4842 | **0.4975** | 0.4767 | 0.4841 |
| MiFish | 0.7340 | **0.7435** | 0.7421 | 0.7262 |
| Berry | 0.6961 | 0.6896 | **0.7037** | 0.7026 |
| Ac16s | 0.7598 | **0.7693** | 0.7616 | 0.7653 |

Table S2 : Training hyperparameters for the transductive pre-training and supervised fine-tuning phases

| Model | lr | Optimizer | Batch size | Epochs | Scheduler | Best model criterion |
| --- | --- | --- | --- | --- | --- | --- |
| BouillaBERT_trans | 2e-5 | AdamW | 16 | Max 150  earlystopping | Linear | CrossEntropyLoss on Masked Token |
| Supervised fine-tuning | 2e-5 | AdamW | 16 | Max 15 w/  earlystopping | Cosine with restarts | Evaluation family macro-F1 |
| BERTax | 1e-5 | AdamW | 8 | Max 20 w/  earlystopping | None | Evaluation family loss |
| DNAGPT | 3e-5 | AdamW | 8 | Max 20 w/  earlystopping | Cosine with warm up | Evaluation family loss |

Supplementary note S1 : MMseqs2 command line

*commande : mmseqs easy-taxonomy --search-type 3 -e 1e-5 --lca-mode 4, search-type 3 = nucleotide, -e : List matches below this E-value, --lca-mode 4: lowest common ancestor of all equal scoring tophits*

Supplementary figure S1 : Macro averaged recall, precision and F1 optimization curves for Mmseqs2, across all markers
